# Consensus on Control of Risky Nonvariceal Upper Gastrointestinal Bleeding in Taiwan with National Health Insurance

**DOI:** 10.1155/2014/563707

**Published:** 2014-08-14

**Authors:** Bor-Shyang Sheu, Chun-Ying Wu, Ming-Shiang Wu, Cheng-Tang Chiu, Chun-Che Lin, Ping-I Hsu, Hsiu-Chi Cheng, Teng-Yu Lee, Hsiu-Po Wang, Jaw-Town Lin

**Affiliations:** ^1^Institute of Clinical Medicine, National Cheng Kung University Hospital, College of Medicine, National Cheng Kung University, Tainan, Taiwan; ^2^Department of Internal Medicine, National Cheng Kung University Hospital, College of Medicine, National Cheng Kung University, Tainan, Taiwan; ^3^Department of Internal Medicine, Taichung Veterans General Hospital, Taichung, Taiwan; ^4^Department of Internal Medicine, National Taiwan University Hospital, Taipei, Taiwan; ^5^Gastroenterology Endoscopy Center, Chang Gung Memorial Hospital, Linko, Taiwan; ^6^Department of Internal Medicine, Chung Shan Medical University Hospital, Taichung, Taiwan; ^7^Department of Internal Medicine, Tainan Hospital, Kaohsiung Veterans General Hospital, Kaohsiung, Taiwan; ^8^School of Medicine, Fu Jen Catholic University, No. 510 Zhongzheng Road, Xinzhuang District, New Taipei City 24205, Taiwan

## Abstract

*Background and Aims*. To compose upper gastrointestinal bleeding (UGIB) consensus from a nationwide scale to improve the control of UGIB, especially for the high-risk comorbidity group. * Methods*. The steering committee defined the consensus scope to cover preendoscopy, endoscopy, postendoscopy, and overview from Taiwan National Health Insurance Research Database (NHIRD) assessments for UGIB. The expert group comprised thirty-two Taiwan experts of UGIB to conduct the consensus conference by a modified Delphi process through two separate iterations to modify the draft statements and to vote anonymously to reach consensus with an agreement ≥80% for each statement and to set the recommendation grade. * Results*. The consensus included 17 statements to highlight that patients with comorbidities, including liver cirrhosis, end-stage renal disease, probable chronic obstructive pulmonary disease, and diabetes, are at high risk of peptic ulcer bleeding and rebleeding. Special considerations are recommended for such risky patients, including raising hematocrit to 30% in uremia or acute myocardial infarction, aggressive acid secretory control in high Rockall scores, monitoring delayed rebleeding in uremia or cirrhosis, considering cycloxygenase-2 inhibitors plus PPI for pain control, and early resumption of antiplatelets plus PPI in coronary artery disease or stroke. * Conclusions*. The consensus comprises recommendations to improve care of UGIB, especially for high-risk comorbidities.

## 1. Introduction

Upper gastrointestinal bleeding (UGIB) is a highly prevalent and potentially fatal condition worldwide [1, 2]. The incidence of UGIB has an increasing trend in elderly people with comorbid illnesses and in users of nonsteroid anti-inflammatory drugs (NSAIDs) [1, 2]. The recommendations from previous UGIB guidelines and reviews have resulted in improvements in patients care and outcomes [3–5]. The international consensus recommendations on the management of patients with nonvariceal UGIB were updated in 2010 with a substantial expansion [1]. Due to concerns about Asia-Pacific regional differences in patient characteristics and healthcare systems as compared to developed Western countries, some specific strategies have been revised [2]. However, there remains evident diversity in the availability of medications and endoscopy facilities within the Asia-Pacific region.

In Taiwan, endoscopy and the therapeutic modalities are readily available on a nationwide scale. Moreover, the National Health Insurance program, which covers more than 99% of the entire population of Taiwan, provides full support for medication and endoscopy for UGIB. Therefore, there is a need to refresh the current consensus for patient care of peptic ulcer bleeding. Although some recommendations in this consensus are based on local data extracted from the National Health Insurance program, these recommendations address many important issues, for example, comorbidity, also emerging in other countries [6]. Because patients are now older and sicker than before, the consensus statements can be applied in general to improve evolution in health care due to the aging population worldwide.

Owing to the National Health Insurance Research Database (NHIRD) which covers more than 23 million residents over more than 15 years, the current consensus has provided strong evidence of its validity in a nationwide cohort setting for UGIB. To collect recommendations from a nationwide scale to improve the outcomes of UGIB, especially to improve the care for the high-risk group, is a novel project.

## 2. Methods

### 2.1. Scope Setting and Preparation Structure of Consensus by a Steering Committee

To establish the expert consensus of UGIB in Taiwan, the steering committee was initiated by J. T. Lin, chaired by B. S. Sheu, and cochaired by C. Y. Wu along with seven other opinion leaders from the Gastroenterological Society of Taiwan (M. S. Wu, C. T. Chiu, C. J. Lin, P. I. Hsu, H. C. Cheng, T. Y. Lee, and H. P. Wang). The steering committee defined the scope sessions of the consensus, searched for and reviewed the literature, formulated the draft statements, and defined the statement evidence level.

### 2.2. Literature Search and Review to Address the Draft Statements with Evidence Level Grading

The literature searches included Medline, Embase, the Cochrane Central Register of Controlled Trial, and ISI Web of Knowledge, with manual searches of bibliographies of key articles and proceedings of abstracts of major gastroenterology conferences held over the past 7 years. The keywords used in the search included gastrointestinal bleeding, peptic ulcer, proton pump inhibitor, upper gastrointestinal endoscopy, rebleeding, and mortality.

The members of the steering committee summarized the findings into the four scope sessions of this consensus: the first three sessions were ranked in order by patient-centric time-framed allocations from preendoscopy, endoscopy, and postendoscopy assessments, and the last session commented on the particular scenario of an overview of UGIB from the Taiwan NHIRD. Based on the review of the literature, the draft statements of the consensus were established by the session leader(s) of each scope session. For each statement, the level of evidence was defined according to modified grading of the Oxford Centre for Evidence-Based Medicine Levels of Evidence (March, 2009) [7]. The draft statements were refined at the steering committee meeting held in Tainan during May 2013.

### 2.3. Expert Group Process to Achieve Agreement of Statement and Grading of Recommendation

The expert group of the Taiwan UGIB consensus comprised a total of 32 experts, including 10 members in the steering committee and 22 members who accepted the invitation of the steering committee. The draft statements from the four session groups were sent to all experts, together with pertinent literature before the consensus meeting in Taichung in July 2013.

During the two-day consensus meeting, for each draft statement from the four scope sessions, the supporting evidence from the keynote literature summary by the steering committee was presented serially in order from preendoscopy, endoscopy, and postendoscopy to NHIRD assessments. Based on a modified Delphi process through two separate iterations, all participants voted anonymously for the first round of statements and modified the statements by discussion. The modified statements were followed by a second round of voting with electronic keypads until a consensus was reached at the agreement percentage of ≥80%. If the agreement was less than 80%, the statement was rejected.

The expert members also discussed the level of evidence suggested by the steering committee and then provided grading of the recommendation level by voting for each statement. The grading of recommendation into 4 grades from A to D was applied as in the Asia-Pacific working group consensus for UGIB [2]. The level of recommendation was defined as the grade with the highest number of votes of the expert group members. The conferences were underwritten by unrestricted grants from the Gastroenterological Society of Taiwan. Mandatory written disclosures of financial conflict of interests within the period of three years before the meetings were obtained from all experts before voting.

## 3. Consensus Statements

### 3.1. Section I: Preendoscopy Assessment

*Statement I-1. For specific comorbid patients with uremia or coronary artery diseases, to raise hematocrit at least >30% shall be beneficial* (agreement: 94%, level of evidence: 1b, and recommendation: A).

For patients who present with acute bleeding, hemoglobin (Hb) concentration < 7 g/dL is an indication for blood transfusion. For those with Hb concentration > 10 g/dL, blood transfusion is rarely indicated [1]. Among patients with bleeding from a peptic ulcer, further bleeding risk is lower in the strategy with threshold transfusion of Hb < 7 g/dL than in the strategy with Hb < 9 g/dL [8].

Special consideration should be given for blood transfusions in UGIB patients with comorbid diseases including uremia and acute myocardial infarction to keep the hematocrit level above 30% [9, 10]. The platelet-mediated hemorrhagic tendency in uremia may be managed successfully by raising hematocrit to above 30% [11].

*Statement I-2. The preendoscopy Rockall score is a useful tool to identify high-risk patients who need further endoscopic therapy and radiologic and surgical interventions* (agreement: 90%, level of evidence: 2b, and recommendation: B).

Rockall scoring system combining both clinical and endoscopic variables may identify patients who are given early discharge or outpatient management who are in need of more aggressive treatment interventions, including endoscopy, and who have further bleeding or death [12]. In predicting the need for endoscopic therapy, the Glasgow-Blatchford score may be more useful in detecting which patients need clinical intervention than the preendoscopic Rockall score [13, 14]. However, only 1% to 5% of cohort cases have Glasgow-Blatchford score of 0 to indicate that they are at low risk and intervention is not required [14, 15]. So the expert discussions preserve the statement to be more focused on Rockall score and suggest there should be a need of local validations for the Glasgow-Blatchford score in future. 

*Statement I-3. Preendoscopic intravenous proton pump inhibitor can enhance resolution of stigmata of bleeding and decrease the need of endoscopic therapy, though it cannot replace the urgent endoscopy* (agreement: 97%, level of evidence: 1a, and recommendation: A).

The use of intravenous proton pump inhibitors (PPI) before endoscopy can reduce endoscopic therapy at index endoscopy, but it does not improve the clinical outcome of UGIB including rebleeding, surgery, or mortality [16, 17]. Nevertheless, endoscopy should generally be conducted within 24 hours for UGIB [18, 19], and the preendoscopy administration of PPI should not delay or replace urgent endoscopy for UGIB.

### 3.2. Section II: Endoscopy Assessment

*Statement II-1. Stigmata of recent hemorrhage (SRH) of bleeding peptic ulcer can predict the risk of rebleeding and guide management decisions. Forrest classification is commonly used to describe SRH* (agreement: 100%, level of evidence: 2b, and recommendation: A).

The stigmata of recent hemorrhage (SRH) are now widely used to record the endoscopic finding of bleeding peptic ulcers with the classification of Forrest et al., to disclose recurrent bleeding rates [20, 21], and to guide endoscopic hemostasis [22–25] and time to discharge [26–28]. For example, a visible vessel has average 43% rebleeding risk [21], needs endoscopic hemostatic therapies [24], and takes four days to disappear [26]. 

*Statement II-2. Endoscopic therapy is recommended to be provided for patients with high-risk lesions, such as active spurting, oozing bleeding, or a nonbleeding visible vessel (Forrest Ia, Ib, or IIa)* (agreement: 100%, level of evidence: 1a, and recommendation: A).

Meta-analyses of randomized controlled trials confirmed the benefits of endoscopic hemostatic therapies to arrest active bleeding or decrease recurrent bleeding for high-risk bleeding peptic ulcers (such as Forrest Ia, Ib, or IIa lesions) [22–25]. Endoscopic hemostatic therapies including injection, thermal therapy, and a combination are better than pharmacotherapy only to control peptic ulcer recurrent bleeding [24, 25].

*Statement II-3. Endoscopic therapy may be considered for ulcers with adherent clots (Forrest IIb)* (agreement: 100%, level of evidence: 1a, and recommendation: A).

A meta-analysis suggested that endoscopic therapy is superior to medical therapy and to decrease recurrent bleeding and surgery but without improvement in mortality [29]. However, another meta-analysis did not show significant benefit in any clinical outcomes [24]. Because these studies had the variable definition of adherent clots and different results, what to do with clots remains inconclusive.

*Statement II-4. Endoscopic therapy is not routinely recommended to ulcers with a flat pigmented spot or a clean base (Forrest IIc or III)* (agreement: 93%, level of evidence: 2b, and recommendation: A).

The rates of recurrent bleeding may be as low as 5%~10% in bleeding ulcers with a clean base and flat pigment spot without endoscopic therapy [21], and they are thus not in need of endoscopic therapy. 

*Statement II-5. Epinephrine injection therapy is recommended to be combined with a second modality* (agreement: 90%, level of evidence: 1a, and recommendation: A).

Endoscopic injection of epinephrine is less effective than other monotherapies to prevent recurrent ulcer bleeding [24, 25]. By combining epinephrine injections with a second modality (such as thermal coagulation, fibrin glue, or hemoclip), the outcome of UGIB, including further bleeding and the need for surgery, may be much improved [22–25, 30].

### 3.3. Section III: Postendoscopy Assessment

*Statement III-1. Patients with bleeding peptic ulcers are recommended to be treated with intravenous high dose or nonhigh dose of proton pump inhibitors, as bolus or continuous infusion for 72 hours after successful endoscopic therapy* (agreement: 97%, level of evidence: 1a, and recommendation: A).

An intravenous PPI administration can improve control of peptic ulcer bleeding after endoscopic therapy [31, 32]. A nonhigh dose regimen can still be as efficacious as a high dose regimen of PPI (such as esomeprazole at least 8 mg/hr intravenous infusion for 72 hr) to control recurrent peptic ulcer bleeding [32–34]. Nevertheless, a recent Cochrane review showed that low quality evidence did not exclude either a potential reduction or an increase in outcomes including rebleeding, surgery, mortality, and repeated endoscopic hemostatic treatment, with high dose compared to nonhigh dose proton pump inhibitor regimens [35].

*Statement III-2. Oral proton pump inhibitors could be an alternative treatment to intravenous infusion after successful endoscopic hemostasis for low-risk peptic ulcer bleeding* (agreement: 87%, level of evidence: 1b, and recommendation: B).

The hospital stay, need for blood transfusion, recurrent bleeding, and mortality are similar for oral high dose PPI and intravenous PPI infusion after endoscopic hemostasis among low-risk patients, whose Rockall scores <6 or American Society of Anesthesiologists class I or II [36, 37]. 

*Statement III-3. For NSAID users with previous peptic ulcer bleeding, either nonselective NSAID to plus PPI or COX-2 inhibitor alone can reduce the recurrent peptic bleeding. Otherwise, COX-2 inhibitor plus PPI may offer better gastroduodenal protection* (agreement: 97%, level of evidence: 1b, and recommendation: A).

For the users of nonsteroid anti-inflammatory drugs (NSAIDs) with a previous history of peptic ulcer bleeding, either a cycloxygenase-2 (COX-2) inhibitor or a nonselective NSAID plus PPI can reduce the recurrent bleeding [38]. The combination of a COX-2 inhibitor and PPI can achieve nearly no recurrence rate of peptic ulcer bleeding [39].

*Statement III-4. Patients with comorbidities or poor nutrition status have higher incidence of peptic ulcer diseases and recurrent bleeding* (agreement: 100%, level of evidence: 2b, and recommendation: B).

Nosocomial bleeding [40] and the presence of comorbidities [12, 41] including uremia [34, 42, 43], liver cirrhosis [41, 44], chronic obstructive pulmonary disease [34], and poor nutrition status as hypoalbuminemia [45] are the significant factors to have a higher incidence of peptic ulcer disease or recurrent bleeding. The domestic data suggested the effect of 3-day PPI infusion after therapeutic endoscopy was limited to prevent recurrence bleeding in patients with comorbidities [45, 46]. Extending the duration of intravenous PPI infusion to 7 days or doubling the dose of oral PPI as twice daily after 3-day intravenous infusion can improve the control of recurrent bleeding in such high-risk populations [47, 48]. This implies that more aggressive acid control is necessary for high-risk patients who are defined by Rockall score ≥6 [48].

*Statement III-5. In bleeding ulcer patients who require low-dose aspirin therapy for cardiovascular prophylaxis, aspirin plus PPI should be restarted as soon as possible, once hemostasis can be achieved or cardiovascular risks outweigh gastrointestinal risks* (agreement: 97%, level of evidence: 1b, and recommendation: A).

It is reasonable to stop the antiplatelet therapy during acute ulcer bleeding [49]. Once hemostasis is achieved, early resumption of antiplatelet agents with PPI at 3-5 days after the last dose can be suitable [50, 51]. For the long-term prevention of peptic ulcer bleeding, cotherapy with PPI is suggested for aspirin or clopidogrel users [52–55]. The COGENT trail confirmed a reduction in gastrointestinal bleeding risk without increase in cardiovascular events when clopidogrel was coprescribed with omeprazole [56].

*Statement III-6. The second-look endoscopy is not routine for all patients but can be reserved for the high-risk patients* (agreement: 100%, level of evidence: 1a, and recommendation: B).

There are no proven benefits by second-look endoscopy and, considering the availability of high dose PPIs, the second-look endoscopy may be reserved for high-risk patients [57, 58]. A meta-analysis showed that routine second-look endoscopy in peptic ulcer bleeding might be effective in high-risk patients, including those with hemodynamic instability, active bleeding, large ulcers, ulcer of posterior wall of bulb, and more or active comorbidities [1, 58]. Therefore, there is a pressing need for further studies to elucidate the role and, moreover, the selection criteria of second-look endoscopy. This is the reason why the expert members had 100% agreement but only B recommendation.

### 3.4. Section IV: Special Scenario of an Overview of UGIB from the NHIRD

*Statement IV-1. Taiwan NHIRD researches identify the high-risk populations of peptic ulcer bleeding and recurrent bleeding, including liver cirrhosis, end stage renal disease, probably chronic obstructive pulmonary disease, and type II diabetes* (agreement: 100%, level of evidence: 2b, and recommendation: B).

A number of nationwide studies using the NHIRD in Taiwan have identified several populations at risk of peptic ulcer bleeding, including those with liver cirrhosis, end stage renal disease, type II diabetes, chronic obstructive pulmonary disease, age > 65 years, male gender, hypertension, heart failure, history of peptic ulcer disease, and chronic users of NSAIDs [59–63]. These underlying comorbidities may serve as independent risk factors of the recurrent ulcer bleeding [46, 64, 65]. The effect of life-long antisecretory medications in prevention of peptic ulcer recurrence in high-risk populations represents an important topic for future research.

*Statement IV-2. Taiwan NHIRD researches identify NSAID and high-affinity serotonin reuptake inhibitors increase the risk of peptic ulcer bleeding* (agreement: 97%, level of evidence: 3b, and recommendation: B).

The nonselective NSAIDs are significantly associated with a higher risk of UGIB [66, 67]. Selective serotonin reuptake inhibitors also predispose to recurrent bleeding of UGIB [68, 69]. Concomitant use of antisecretory medications may therefore be suggested. 

*Statement IV-3. Taiwan NHIRD researches support the fact that H. pylori eradication reduces peptic ulcer diseases and the risk of gastric cancer for patients with peptic ulcer diseases* (agreement: 90%, level of evidence: 2b, and recommendation: B).

Anti-*H. pylori *therapy given within 6 months of ulcer diagnosis can reduce the ulcer events [70, 71] and prevent bleeding recurrence [72]. An increased risk of gastric cancer correlates with a late eradication beyond one year in patients with peptic ulcer. Early* H. pylori *eradication is therefore suggested as an independent protective factor to reduce the risk of gastric cancer [73]. This result is compatible with a large-scale study and meta-analysis, showing that eradication of* H. pylori* decreases the development of gastric cancer only among those without precancerous lesion [74, 75].

## 4. Dissemination Strategies and Legal Issues

These statements are based on the best available evidence to pursue better quality of care and will be updated per 5 years. They are not suitable for deciding standard of care in specific cases. This consensus statement will be disseminated by (1) presentations given at the annual society meeting of Taiwan Digestive Week in 2013; (2) possible release of copies of these statements in electronic and paper format to national societies/associations of gastroenterologists for their iterations; (3) release on the website of our society link.

## Abbreviations

COX-2: Cycloxygenase-2
NHIRD: National Health Insurance Research Database
NSAIDs: Nonsteroid anti-inflammatory drugs
NTT: Number needed for treatment
PPI: Proton pump inhibitor
RR: Relative risk
SRH: Stigmata of recent hemorrhage
UGIB: Upper gastrointestinal bleeding.
